# Association of the RYR3 gene polymorphisms with atherosclerosis in elderly Japanese population

**DOI:** 10.1186/1471-2261-14-6

**Published:** 2014-01-14

**Authors:** Chenxi Zhao, Shinobu Ikeda, Tomio Arai, Makiko Naka-Mieno, Noriko Sato, Masaaki Muramatsu, Motoji Sawabe

**Affiliations:** 1 Department of Molecular Epidemiology, Medical Research Institute, Tokyo Medical and Dental University, 2-3-10 Kanda-Surugadai, Chiyoda-ku, Tokyo 101-0062, Japan; 2 Department of Pathology, Tokyo Metropolitan Geriatric Hospital, Tokyo, Japan; 3 Department of Medical Informatics, Center of Information, Jichii Medical University, Tochigi, Japan; 4 Section of Molecular Pathology, Graduate School of Health Care Sciences, Tokyo Medical and Dental University, Tokyo, Japan

**Keywords:** Atherosclerosis, Polymorphism, Ryanodine receptor 3, Japanese

## Abstract

**Background:**

The Ryanodine receptor 3 gene (*RYR3*) encodes an intracellular calcium channel that mediates the efflux of Ca^2+^ from intracellular stores. Two single-nucleotide polymorphisms (SNPs) in the *RYR3* gene have been shown to associate with stroke (*rs877087*) and carotid intima-media thickness (*rs2229116*) in two independent genome-wide association studies (GWAS) in Caucasian. We investigated the effect of these two SNPs as well as the 31.1 kilobases spanning region on atherosclerosis in Japanese population.

**Methods:**

Atherosclerotic severity was assessed by carotid artery (n = 1374) and pathological atherosclerosis index (PAI) (n = 1262), which is a macroscopic examination of the luminal surfaces of 8 systemic arteries in consecutive autopsy samples. 4 tag SNPs in the 31.1 Kb region, *rs877087*, *rs2132207*, *rs658750* and *rs2229116*, were genotyped and haplotypes were inferred to study the association with atherosclerotic indices.

**Results:**

*rs877087* and *rs2229116* were associated with PAI (OR = 2.07 [1.04-4.12] (95% CI), *p* = 0.038; and OR = 1.38 [1.02-1.86], *p* = 0.035, respectively). *rs2229116* was also associated with common carotid atherosclerosis (OR = 1.45 [1.13-1.86], *p* = 0.003). The risk allele of *rs2229116* was opposite from the original report. The haplotype block of this 31.1 Kb region was different between Caucasian and Japanese. Haplotype analysis revealed that only TAGG haplotype was associated with PAI (OR = 0.67 [0.48-0.94], *p* = 0.020) and atherosclerosis of common carotid artery (OR = 0.75 [0.58-0.98], *p* = 0.034).

**Conclusion:**

*rs877087* and *rs2229116* of *RYR3* gene are associated with atherosclerosis severity in Japanese. The functional difference caused by *rs2229116* needs to be investigated.

## Background

Atherosclerosis is currently thought to be an inflammatory disease with deposits of cholesterol and inflammatory cells in the arterial wall [1-3]. In the later stages, thrombosis forms, and this leads to fatal events, like myocardial and cerebral infarctions [4,5]. The etiology of atherosclerosis is multi-factorial, in that it includes various genetic and environmental factors [6,7]. It is known that genetic factors play a pivotal role in atherosclerosis, but the influence of specific genes on susceptibility is not fully understood [8-10]. The discovery of novel risk factors for atherosclerosis may lead to efficient prevention and new therapeutic targets for this fatal disease.

Recently, two independent genome-wide association studies (GWAS) of atherosclerotic diseases in Caucasians pointed to two SNPs, closely resided in the 31.1 Kb (intron 14 ~ exon 19) region of the *RYR3* gene [11,12]. *RYR3* gene is located on chromosome 15q14-q15, and encode a large intracellular homotetrameric protein (> 2MDa) that comprises 4780 amino acids [13-15]; RYR3 protein resides on the sarcoplasmic reticulum membrane and releases Ca2+ from intracellular stores to regulates intracellular calcium concentration [16,17]. The RyR3-deficient mice lack calcium regulation properties in arterial smooth muscle cells, which lead to dysregulation of arterial tone [18]. In human arterial endothelial cells, Ca2+ release mediated by RYR3, but not by the RYR1 and RYR2, plays a role in endothelial vasodilation [19]. Thus, it is conceivable that RYR3 is a good candidate for atherosclerosis susceptible gene. The *rs877087* polymorphism, which is an intron SNP, was associated with stroke in a Caucasian population from 4 large cohorts (n = 19,602) [11]. The *rs2229116*, which is an Ile to Val non-synonymous SNP, was associated with common carotid intima-media thickness (cIMT), a clinical parameter for subclinical atherosclerosis [20]. This was found in a specific group of Caucasian males infected with HIV and were treated with highly active antiretroviral therapy (n = 177) [12]. Since *rs877087* and *rs2229116* on *RYR3* gene is separated by 31.1 Kb, we hypothesize that this region might confer susceptibility to atherosclerosis, and thus studied this region in detail using tag SNPs and haplotypes.

## Methods

### Subjects

A total of 1536 consecutive autopsy cases of aged patients registered in the JG-SNP database were examined (http://www.tmghig.jp/jg-snp/english/E_top.html) which contains abundant pathological information on atherosclerosis [21]. The autopsies were performed from 1995 to 2004 at the Tokyo Metropolitan Geriatric Hospital in Tokyo, Japan. The distribution of age at death is 65 ~ 102 years old, and the mean is 80.21 ± 8.90 (mean ± SD). The major clinical diagnosis and direct cause of death in this Japanese population was described elsewhere [21]. The study was approved by the ethical committees of the Tokyo Metropolitan Geriatric Hospital and the Tokyo Medical and Dental University.

The methods for the pathological macroscopic evaluation of atherosclerosis severity are published previously [22,23]. Briefly, a total of eight arteries were examined, including the common carotid artery, subclavian artery, aorta, splenic artery, superior mesenteric artery, common iliac artery, external iliac artery, and femoral artery. Assessments were performed by macroscopic examinations of the luminal surface in formalin-fixed arteries. The degree of atherosclerosis had been scored according to the ratio of the atheroma area to the entire intimal area. The scale ranged from 0–8, where 0 = absent, or a ratio less than 1/20; 2 = minimal, or a ratio of 1/20 ~ 1/6; 4 = mild, or a ratio of 1/6 ~ 1/3; 6 = moderate, or a ratio of 1/3 ~ 2/3; and 8 = severe, or a ratio of 2/3 ~ 1. The pathological atherosclerotic index (PAI) was defined as the average degree of atherosclerotic severity in all eight arteries [23]. Since not all eight arteries were evaluated in some samples, the number of PAI records (n = 1262) is less than the number of common carotid artery records (n = 1374).

### Genetic analyses

The *RYR3* polymorphism (*rs877087*, *rs2132207*, *rs658750* and *rs2229116*) was analyzed by the Taqman assay (Applied BioSystems) using specific primers and probes on a Light-Cycler 480 (Roche), according to previously described protocols [24]. The pathological assessments and genotyping were performed in different institutions in a double-blind fashion.

### Statistical analyses

Statistical analyses were performed with the IBM SPSS 19.0.0 statistical software. The associations of genotypes with atherosclerosis severity were tested by multiple logistic regression analyses with adjustment for conventional risk factors. All continuous and discrete variables of atherosclerotic severity were categorized by the cut-point of 75% severity, and divided subjects into Ath(+) group with top 25% severity, and Ath(−) group with severity lower than that, as we did previously [22]. The conventional risk factors were also categorized as follows: gender (male vs. female), hypertension, diabetes mellitus, hyperlipidemia, and history of smoking or drinking (absent vs. present were classified as 0 vs. 1, respectively). P-values less than 0.05 were considered statistically significant, and correction for multiple testing was not considered in this study. Haplotype frequency estimation and haplotype-based associations were performed using PLINK software (http://pngu.mgh.harvard.edu/purcell/plink/) [25]. Hardy–Weinberg equilibrium (HWE) was determined with Fisher’s exact test. Linkage disequilibrium among SNPs was assessed using Haploview software [26].

## Results

### Genetic structure data

**Figure 1:**
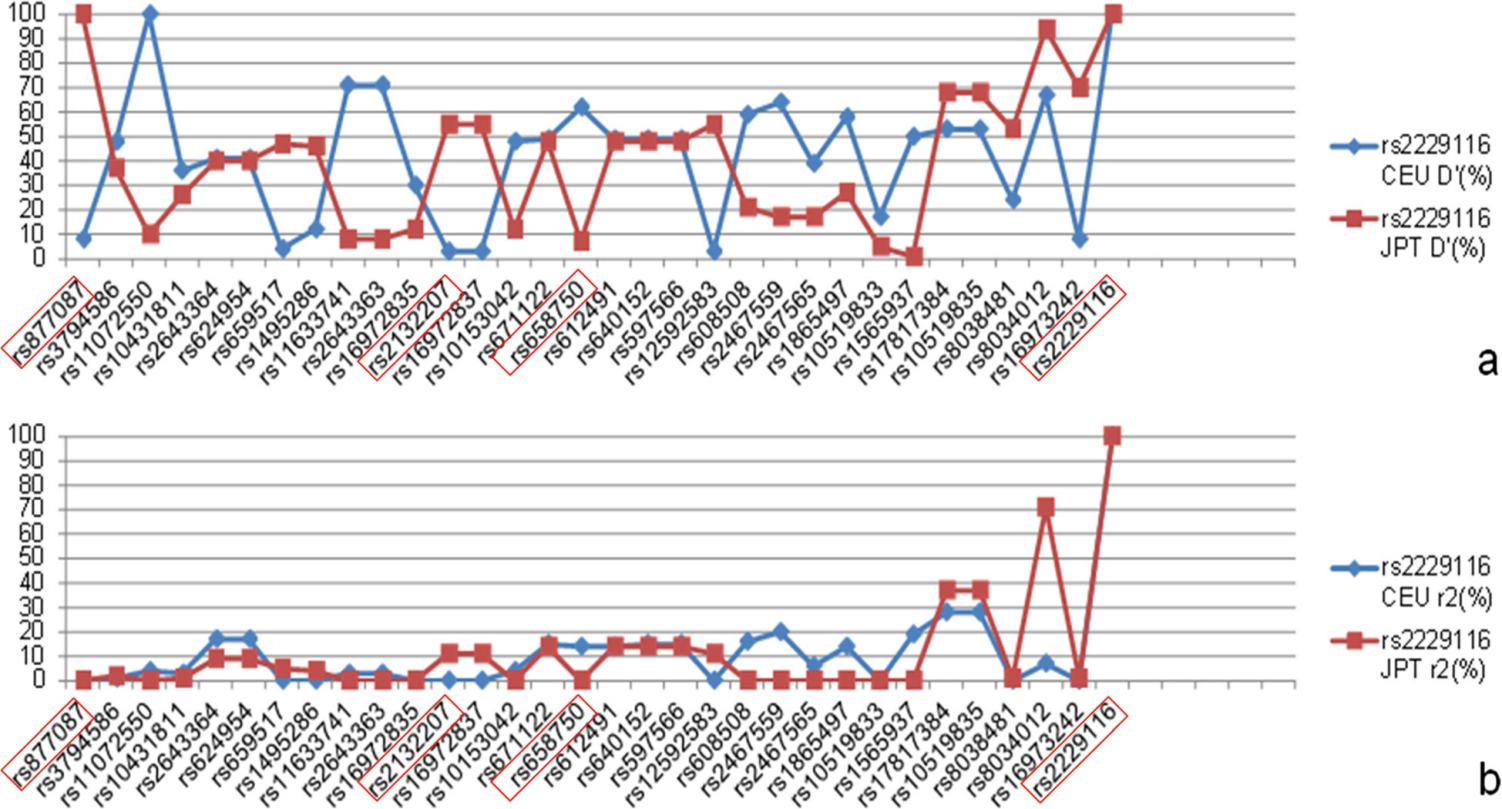
**LD of SNPs between the 31.1 Kb region of *rs877087 *and *rs2229116 *in CEU and JPT. a**: Comparison of D’ values (%) between CEU and JPT. *rs2229116* as reference SNP. Data were derived from HapMap. **b**: Comparison of r^2^ (%) values in this region.

*rs877087* and *rs2229116* is 31.1 Kb apart and the D’ and r^2^ values of this susceptibility region in Japanese and Caucasian were shown in Figure 1. The trends of LD value showed that distinct difference of LD values could be found in several SNP sites between two the populations (Figure 1). In order to investigate this region, two tag SNPs (*rs2132207* and *rs658750*, both are intron SNPs) between *rs877087* and *rs2229116*, were selected by using Haploview software (pairwise tagging, r^2^ threshold ≥ 0.1). Genotype and haplotype data of the four *RYR3* polymorphisms are summarized in Table 1. Five major haplotypes (TGGA, TAGA, TAAA, TAGG and TGGG) from the 4 SNPs could explain near 90% of the genetic diversity constituted by these 4 SNPs in this region (Table 1).

**Table 1:** Genotype and haplotype distribution of the SNPs

**rsSNPs**	**Genotypes, n(%)**			**P-value for HWE test**
rs877087	TT	CT	CC	0.102
	1279(94.2)	76(5.6)	3(0.2)	
rs2132207	GG	AG	AA	0.443
	463(34.2)	668(49.4)	221(16.4)	
rs658750	GG	AG	AA	0.629
	978(72.2)	349(25.8)	28(2.0)	
rs2229116	AA	AG	GG	0.191
	923(66.4)	411(29.5)	57(4.1)	
**Haplotypes**	**Estimated frequencies and number of samples**^**a**^			
TGGA	0.504, n = 681			
TAGA	0.158, n = 214			
TAAA	0.121, n = 164			
TAGG	0.102, n = 138			
TGGG	0.057, n = 77			

^a^Frequencies of haplotypes were estimated using PLINK software.

### Relationship between RYR3 polymorphisms and atherosclerosis severity

Comparisons of atherosclerosis severity in each artery for *rs877087* and *rs2229116* genotypes were performed using Fisher’s exact test, and followed by logistic regression analysis in dominate or recessive models, adjusted by gender, history of hyperlipidemia, hypertension, diabetes mellitus, drinking and smoking (Table 2). *rs877087* T allele persisted significant association with PAI (OR = 2.07, 95% CI = 1.04-4.12, *p* = 0.038) after adjustment for conventional risk factors. Also, *rs2229116* A allele present significant associations with PAI (OR = 1.38, 95% CI = 1.02-1.86, *p* = 0.035) and atherosclerosis in common carotid arteries (OR = 1.45, 95% CI = 1.13-1.86, *p* = 0.003) after adjustment. The two tag SNPs, *rs2132207* and *rs658750*, are not significantly associated with PAI and atherosclerosis in common carotid arteries (Additional file 1: Table S1). Subgroup analysis of sex (male/female) for *rs877087* and *rs2229116* showed that, *rs2229116* A allele present significant associations with PAI and common carotid arteries only in males (Table 2). Additional age subgroup analysis for *rs877087* and *rs2229116* showed that *rs877087* T allele persisted significant association with PAI only in subgroup age above 80; *rs2229116* A allele present significant associations with PAI in subgroup age below 80 and with common carotid arteries in subgroup age above 80 (Additional file 2: Table S2).

**Table 2:** Relationship between RYR3 polymorphisms and atherosclerosis severity >75%

**Arteries**	**Ath (+/−)**	**rs877087 genotypes, n(%)**						**rs2229116 genotypes, n(%)**					
		**TT**	**CT**	**CC**	**TT vs. CT + CC**			**AA**	**AG**	**GG**	**AA vs. AG + GG**		
					***p***^***a***^	**OR(95% CI)**^**b**^	***p***^***b***^				***p***^***a***^	**OR(95% CI)**^**b**^	***p***^***b***^
PAI	+	319(96.4%)	11(3.3%)	1(0.3%)	**0.023**	2.07	**0.038**	241(71.5%)	84(24.9%)	12(3.6%)	**0.008**	1.38	**0.035**
(Total)	-	840(93.2%)	59(6.6%)	2(0.2%)		(1.04–4.12)		593(64.1%)	292(31.6%)	40(4.3%)		(1.02–1.86)	
Common carotid	+	666(94.7%)	35(5.0%)	2(0.3%)	0.214	1.31	0.294	501(69.9%)	190(26.5%)	26(3.6%)	**0.003**	1.45	**0.003**
	-	597(93.5%)	40(6.3%)	1(0.2%)		(0.79–2.15)		412(62.7%)	214(32.6%)	31(4.7%)		(1.13–1.86)	
(Total)													
PAI	+	183(96.3%)	7(3.7%)	0(0%)	0.061	1.87	0.164	140(71.8%)	47(24.1%)	8(4.1%)	**0.042**	1.58	**0.026**
(Male)	-	440(92.8%)	33(7.0%)	1(0.2%)		(0.78–4.52)		315(64.5%)	153(31.4%)	20(4.1%)		(1.06–2.38)	
Common carotid	+	381(94.1%)	23(5.7%)	1(0.2%)	0.533	1.11	0.754	297(71.4%)	103(24.8%)	16(3.8%)	**0.005**	1.63	**0.004**
	-	295(93.9%)	19(6.1%)	0(0%)		(0.57–2.16)		201(62.0%)	108(33.3%)	15(4.7%)		(1.17–2.27)	
(Male)													
PAI	+	136(96.5%)	4(2.8%)	1(0.7%)	0.151	2.39	0.124	101(71.1%)	37(26.1%)	4(2.8%)	0.062	1.23	0.374
(Female)	-	400(93.7%)	26(6.1%)	1(0.2%)		(0.79–7.27)		278(63.6%)	139(31.8%)	20(4.6%)		(0.78–1.93)	
Common carotid	+	285(95.6%)	12(4.0%)	1(0.3%)	0.127	1.55	0.267	204(67.8%)	87(28.9%)	10(3.3%)	0.139	1.25	0.234
	-	302(93.2%)	21(6.5%)	1(0.3%)		(0.72–3.37)		211(63.4%)	106(31.8%)	16(4.8%)		(0.86–1.82)	
(Female)													

^a^p-value from Fisher’s exact test, 1-sided.

^b^p-value and odds ratio from logistic regression test after adjusted by gender, history of hyperlipidemia, hypertension, diabetes mellitus, drinking, and smoking. (Parameter of gender was excluded for adjustment in Male and Female subgroups).

p-values less than 0.05 were marked in bold.

### Atherosclerosis degrees in each *rs2229116* genotype

The tendency from the means of atherosclerosis degree in three genotypes of *rs2229116*, showed that risk allele is the A allele in both PAI and common carotid arteries in our samples (Table 3). In common carotid arteries, statistically significant *p*-values were observed from ANOVA test (*p* = 0.030) and from linear regression test (*p* = 0.010), indicating that there is significant linear relationship between degree of atherosclerosis and the *rs2229116* polymorphism. Also, in PAI, the same trend was seen with regard to the genotype but it was not statistically significant (*p* = 0.060) (Table 3).

**Table 3:** Means of atherosclerosis degrees in rs2229116 genotype groups

**Arteries**	**rs2229116 genotypes, mean(n)**					
	**AA**	**AG**	**GG**	**Total**	***p***^***a***^	***p***^***b***^
Common carotid	7.18 (913)	6.63 (404)	6.42 (57)	6.99 (1374)	**0.030**	**0.010**
PAI	4.29 (834)	4.15 (376)	3.97 (52)	4.23 (1262)	0.171	0.060

^a^p value from ANOVA test for means of three genotype groups.

^b^p value from linear regression test for means of three genotype groups.

p-values less than 0.05 were marked in bold.

### Relationships between RYR3 haplotypes and atherosclerosis severity

From haplotype linear regression test, positive beta values were observed in the TGGA haplotype group in common carotid arteries (beta = 0.34, *p* = 0.032) and observed in the TAAA haplotype group in PAI (beta = 0.50, *p* = 0.048). Also, a negative beta value was observed in TAGG haplotype group in common carotid arteries (beta = −0.70, *p* = 0.007) (Table 4). Logistic regression test of haplotype association was performed for atherosclerosis severity >75%. In TAGG haplotype group, significant preventive odds ratio were observed in common carotid arteries (*p* = 0.034, OR = 0.75; 95% CI, 0.58-0.98), and in PAI (*p* = 0.020, OR = 0.67; 95% CI, 0.48-0.94) (Table 4).

**Table 4:** Haplotype-based association test for linear and logistic regression

	**Common carotid**				**PAI**			
**Haplotype**	**BETA**^**a**^	***p***^***a***^	**OR(95% CI)**^**b**^	***p***^***b***^	**BETA**^**a**^	***p***^***a***^	**OR(95% CI)**^**b**^	***p***^***b***^
TGGA	0.34	**0.032**	1.16(0.99–1.36)	0.071	−0.18	0.292	1.07(0.89–1.28)	0.464
TAGA	0.27	0.194	1.12(0.90–1.40)	0.314	−0.11	0.611	1.15(0.90–1.47)	0.265
TAAA	−0.25	0.296	1.02(0.75–1.39)	0.901	0.50	**0.048**	1.19(0.91–1.55)	0.201
TAGG	−0.70	**0.007**	0.75(0.58–0.98)	**0.034**	−0.14	0.603	0.67(0.48–0.94)	**0.020**
TGGG	−0.61	0.109	0.61(0.41–0.90)	**0.013**	−0.09	0.813	0.75(0.47–1.22)	0.247

^a^BETA and p-value from haplotype-based linear regression test.

^b^Odds ratio and p-value from haplotype-based logistic regression test for atherosclerosis serverity > 75%.

p-values less than 0.05 were marked in bold.

## Discussion

The previous GWAS showed that the *RYR3* gene polymorphisms *rs877087* and *rs2229116* was associated with stroke and cIMT, respectively [11,12]. In this study, we investigated these two SNPs on *RYR3* gene and the 31.1 Kb region in between employing elderly Japanese. We observed that both *rs877087* and *rs2229116* was associated with PAI. Our study suggested that the atherosclerosis risk allele is T for *rs877087*, as the same risk allele from the GWAS of stroke. It should be noted that the minor allele frequency of *rs877087* is 0.035 for C allele in Japanese, and 0.42 for T allele in Caucasian according to the HapMap data. *rs2229116* was associated with PAI and atherosclerosis severity in common carotid after adjusted for conventional risk factors (Table 2). However, our risk allele was opposite from the original paper [12], in that, the GWAS for cIMT the risk allele was G, but in our study it was A (Table 3). The LD patterns between *rs877087* and *rs2229116* from HapMap data showed great differences between CEU and JPT (Figure 1 and Additional file 3: Figure S1 and Additional file 4: Figure S2). As reference to *rs2229116*, the LD values of two populations showed large difference (ΔD’ > 0.5) in several SNP sites, including the 2 tag SNPs (*rs2132207* and *rs658750*), suggesting that the haplotypes within this region may be different between CEU and JPT (Figure 1). Therefore, different haplotypes might account for the different observation between Japanese and European. In order to further investigate the region between the two reported SNPs, we further selected 2 tag SNPs (*rs2132207* and *rs658750*) and build haplotypes (Table 1). Haplotype linear regression analyses revealed that the carriers of TAGG haplotype had a decrease trend of atherosclerosis severity in both common carotid arteries and PAI. Also, an increase trend was observed in TGGA haplotype in common carotid arteries and TAAA haplotypes in PAI, respectively (Table 4). In haplotype logistic regression model for atherosclerosis severity trait, we observed TAGG haplotype had a preventive effect in both common carotid arteries and PAI (Table 4). Although we tried to explain the opposite effect in Japanese and Caucasian, using trans-ethnic fine mapping, and selected these two tag SNPs which gave a significant difference in LD value between two ethnics, we could not explain the association with either of the SNP. Nevertheless, taken together the results of haplotype analysis, it is likely that the non-synonymous polymorphism *rs2229116* (Val/Ile) itself may be the primary determinant for the observed associations (Table 4).

The *rs2229116* SNP causes amino acid substitution (Ile to Val) at the 731st amino acid site of RYR3. However, the functional consequence has not yet been investigated. RYR3 have a major role in calcium signaling in the vasculation and thus is a good candidate gene in atherosclerosis pathogenesis.

Along with the function of RYR3 in vascular cells, recent studies have found that RYR3 have function in T cells, which is involved in the inflammation process of atherosclerotic lesions [27,28]. RYR3 appear to regulate Ca2+ signaling in T lymphocytes [29], in which, calcium signaling is essential for its activation and differentiation [30]. The association of T cell activation markers and carotid arterial stiffness was also reported in a group of HIV-infected women [31]. These lines of evidence suggest that, intracellular calcium mobilization mediated by RYR3 may be involved in atherosclerosis process through its common participants, such as arterial smooth muscle cells, endothelial cells and T lymphocytes.

Our study has some limitations. The subjects of the present study were consecutive autopsy cases of patients in a geriatric hospital. Therefore, the selection bias and whether the subjects can represent the demographics of Japan should be evaluated. The autopsy rate of the hospital was kept around 40% and those who died suddenly outside the hospital and medicolegal cases were not autopsied for this study. The leading mortality probability at the age of 75 years according the census data in ‘Abridged Life Tables For Japan 2010’ by the Ministry of Health, Labor and Welfare of Japan (http://www.mhlw.go.jp/english/database/db-hw/lifetb10/index.html) were as follows; 25.9% men vs. 16.5% women by malignant neoplasms, 15.2% men vs. 20.3% women by heart diseases, 10.4% men vs. 12.1% women by cerebrovascular diseases, and 15.3% men vs. 12.4% women by pneumonia. Almost all death rates for major diseases at 75 years of age in Tokyo were consistent with our autopsy data, except for the frequency of malignant neoplasms is a little higher and that of cerebrovascular diseases is lower in our autopsy subjects. Since the national health insurance covers the whole population of Japan and in Japan most death occurred in hospitals, the selection bias from socioeconomic difference of the subjects seems minimal. Altogether, the subjects in our study did not differ greatly from the standard elderly residents in Tokyo, and may represent the general population, including young residents, in Japan rather than a selected hospitalized population.

## Conclusions

*RYR3* gene *rs877087* and *rs2229116* polymorphisms are associated with atherosclerosis in elderly Japanese. Functional studies are still needed to identify the causal variation.

## Abbreviations

RYR3: Ryanodine receptor 3 gene; SNP: Single nucleotide polymorphism; GWAS: Genome-wide association studies; PAI: Pathological atherosclerosis index; cIMT: Common carotid intima-media thickness; HWE: Hardy-Weinberg equilibrium.

## Competing interests

The authors declare that they have no competing interests.

## Authors’ contributions

CZ and MM designed the study and wrote the paper. CZ and SI carried out the laboratory experiments and analyzed the data. CZ, NS and MM interpreted the results. TA, MNM and MS were responsible for sample collection, pathological data acquisition, and evaluation. All authors have contributed to, read and approved the final manuscript.

## Pre-publication history

The pre-publication history for this paper can be accessed here:

http://www.biomedcentral.com/1471-2261/14/6/prepub

## Supplementary Material

Additional file 1: Table S1 Relationship between RYR3 polymorphisms (rs2132207 and rs658750) and atherosclerosis severity >75%. Click here for file

Additional file 2: Table S2 Relationship between RYR3 polymorphisms and atherosclerosis severity >75% in age subgroups. Click here for file

Additional file 3: Figure S1 The LD (D’) heatmap of the 31.1 Kb region in CEU. Click here for file

Additional file 4: Figure S2 The LD (D’) heatmap of the 31.1 Kb region in JPT. Click here for file
