# Supplementary material for: Association of the RYR3 gene polymorphisms with atherosclerosis in elderly Japanese population
Source: BMC Cardiovasc Disord. 2014 Jan 14;14:6. doi: 10.1186/1471-2261-14-6 (PMC3898238; doi:10.1186/1471-2261-14-6)
Supplement: Additional file 1: Table S1 — Relationship between RYR3 polymorphisms (rs2132207 and rs658750) and atherosclerosis severity >75%. [file 1471-2261-14-6-S1.doc]

Supplementary Table 1. Relationship between RYR3 polymorphisms (rs2132207 and rs658750) and atherosclerosis severity >75%

| Arteries | Ath (+/-) |  | rs2132207 genotypes, n(%) | | | |  | rs658750 genotypes, n(%) | | | |
| --- | --- | --- | --- | --- | --- | --- | --- | --- | --- | --- | --- |
|  |  |  | AA | AG | GG | *p^a^* |  | AA | AG | GG | *p^a^* |
|  |  |  |  |  |  |  |  |  |  |  |  |
| PAI | + |  | 61(18%) | 155(47%) | 114(35%) | 0.372 |  | 11(3%) | 91(28%) | 229(69%) | 0.111 |
|  | - |  | 147(16%) | 444(50%) | 306(34%) |  |  | 17(2%) | 235(26%) | 647(72%) |  |
| common carotid | + |  | 116(17%) | 341(48%) | 245(35%) | 0.348 |  | 13(2%) | 187(27%) | 503(72%) | 0.445 |
|  | - |  | 105(17%) | 317(50%) | 211(33%) |  |  | 15(2%) | 159(25%) | 461(73%) |  |

a: p-value from Fisher’s exact test, 1-sided.
