# Supplementary material for: Association of the RYR3 gene polymorphisms with atherosclerosis in elderly Japanese population
Source: BMC Cardiovasc Disord. 2014 Jan 14;14:6. doi: 10.1186/1471-2261-14-6 (PMC3898238; doi:10.1186/1471-2261-14-6)
Supplement: Additional file 2: Table S2 — Relationship between RYR3 polymorphisms and atherosclerosis severity >75% in age subgroups. [file 1471-2261-14-6-S2.doc]

Supplementary Table 2. Relationship between RYR3 polymorphisms and atherosclerosis severity >75% in age subgroups

| Arteries | Ath (+/-) |  | rs877087 genotypes, n(%) | | | | | | |  | rs2229116 genotypes, n(%) | | | | | |
| --- | --- | --- | --- | --- | --- | --- | --- | --- | --- | --- | --- | --- | --- | --- | --- | --- |
|  |  | TT | | CT | CC | TT vs CT+CC | | |  | AA | | AG | GG | AA vs AG+GG | | |
|  |  |  |  |  |  | *p^a^* | OR(95%CI)^b^ | *p^b^* |  |  |  |  |  | *p^a^* | OR(95%CI)^b^ | *p^b^* |
| PAI  (age≥ 80) | + | 229(97.1%) | | 6(2.5%) | 1(0.4%) | **0.022** | 3.26  (1.31-8.16) | **0.011** |  | 168(70.0%) | | 63(26.3%) | 9(3.7%) | 0.136 | 1.13  (0.78-1.64) | 0.505 |
|  | - | 406(93.1%) | | 29(6.7%) | 1(0.2%) |  |  |  |  | 291(65.5%) | | 134(30.2%) | 19(4.3%) |  |  |  |
| common carotid  (age≥ 80) | + | 413(95.6%) | | 17(3.9%) | 2(0.5%) | 0.146 | 1.58  (0.80-3.14) | 0.190 |  | 310(70.5%) | | 116(26.4%) | 14(3.2%) | **0.010** | 1.47  (1.04-2.07) | **0.027** |
|  | - | 275(93.5%) | | 19(6.5%) | 0(0.0%) |  |  |  |  | 186(62.0%) | | 99(33.0%) | 15(5.0%) |  |  |  |
| PAI  (age< 80) | + | 90(94.7%) | | 5(5.3%) | 0(0.0%) | 0.407 | 0.98  (0.32-2.96) | 0.967 |  | 73(75.3%) | | 21(21.6%) | 3(3.1%) | **0.012** | 2.06  (1.15-3.69) | **0.015** |
|  | - | 434(93.3%) | | 30(6.5%) | 1(0.2%) |  |  |  |  | 302(62.8%) | | 158(32.8%) | 21(4.4%) |  |  |  |
| common carotid  (age< 80) | + | 253(93.4%) | | 18(6.6%) | 0(0.0%) | 0.514 | 1.05  (0.50-2.21) | 0.905 |  | 191(70.0%) | | 74(26.7%) | 12(4.3%) | 0.080 | 1.37  (0.94-1.99) | 0.102 |
|  | - | 322(93.6%) | | 21(6.1%) | 1(0.3%) |  |  |  |  | 226(63.3%) | | 115(32.2%) | 16(4.5%) |  |  |  |

a: p-value from Fisher’s exact test, 1-sided.

b: p-value and odds ratio from logistic regression test after adjusted by gender, history of hyperlipidemia, hypertension, diabetes mellitus, drinking, and smoking.
