# Supplementary figures and images for: Association of the RYR3 gene polymorphisms with atherosclerosis in elderly Japanese population
Source: BMC Cardiovasc Disord. 2014 Jan 14;14:6. doi: 10.1186/1471-2261-14-6 (PMC3898238; doi:10.1186/1471-2261-14-6)

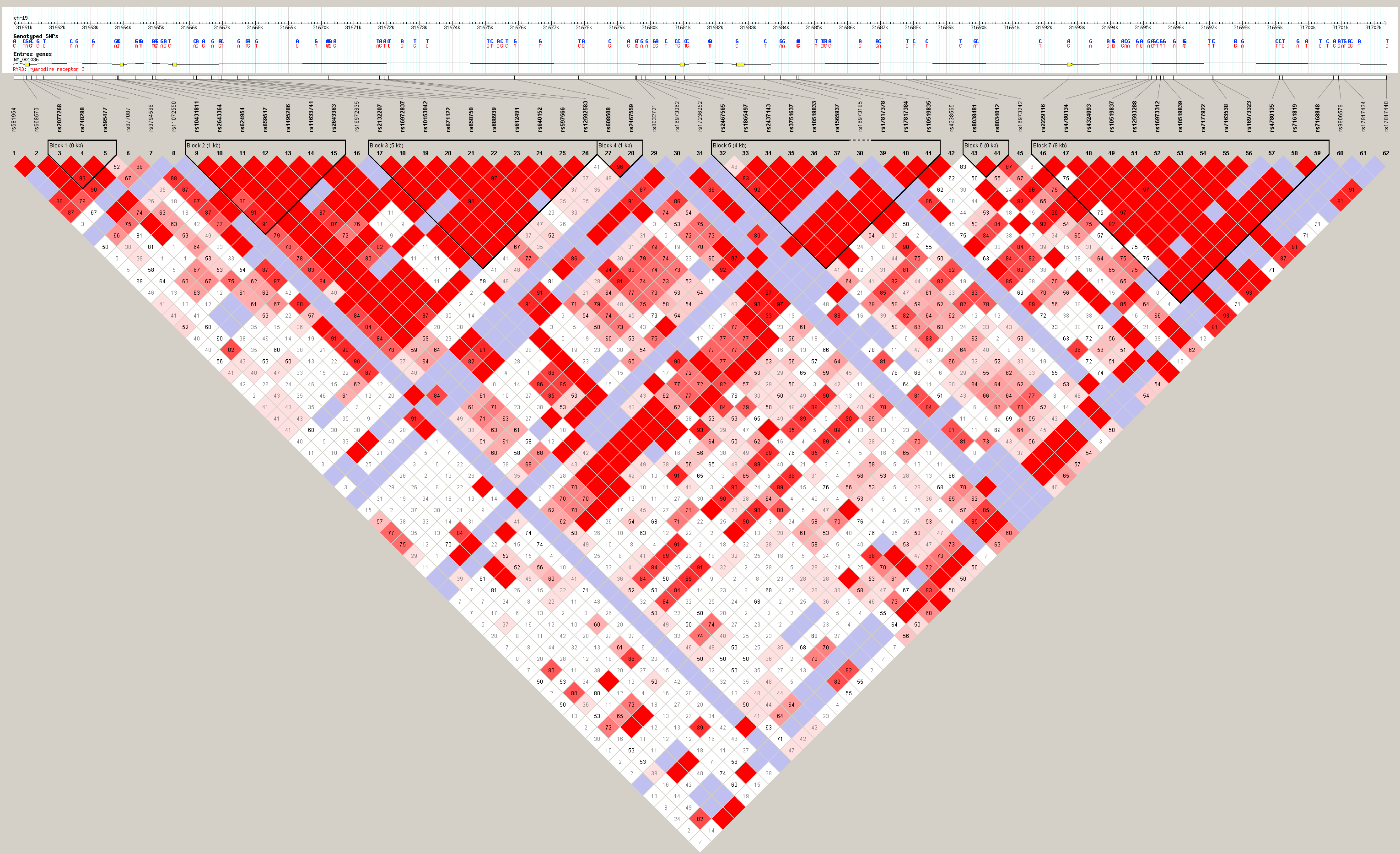

Supplement: Additional file 3: Figure S1 — The LD (D’) heatmap of the 31.1 Kb region in CEU. [file 1471-2261-14-6-S3.png]

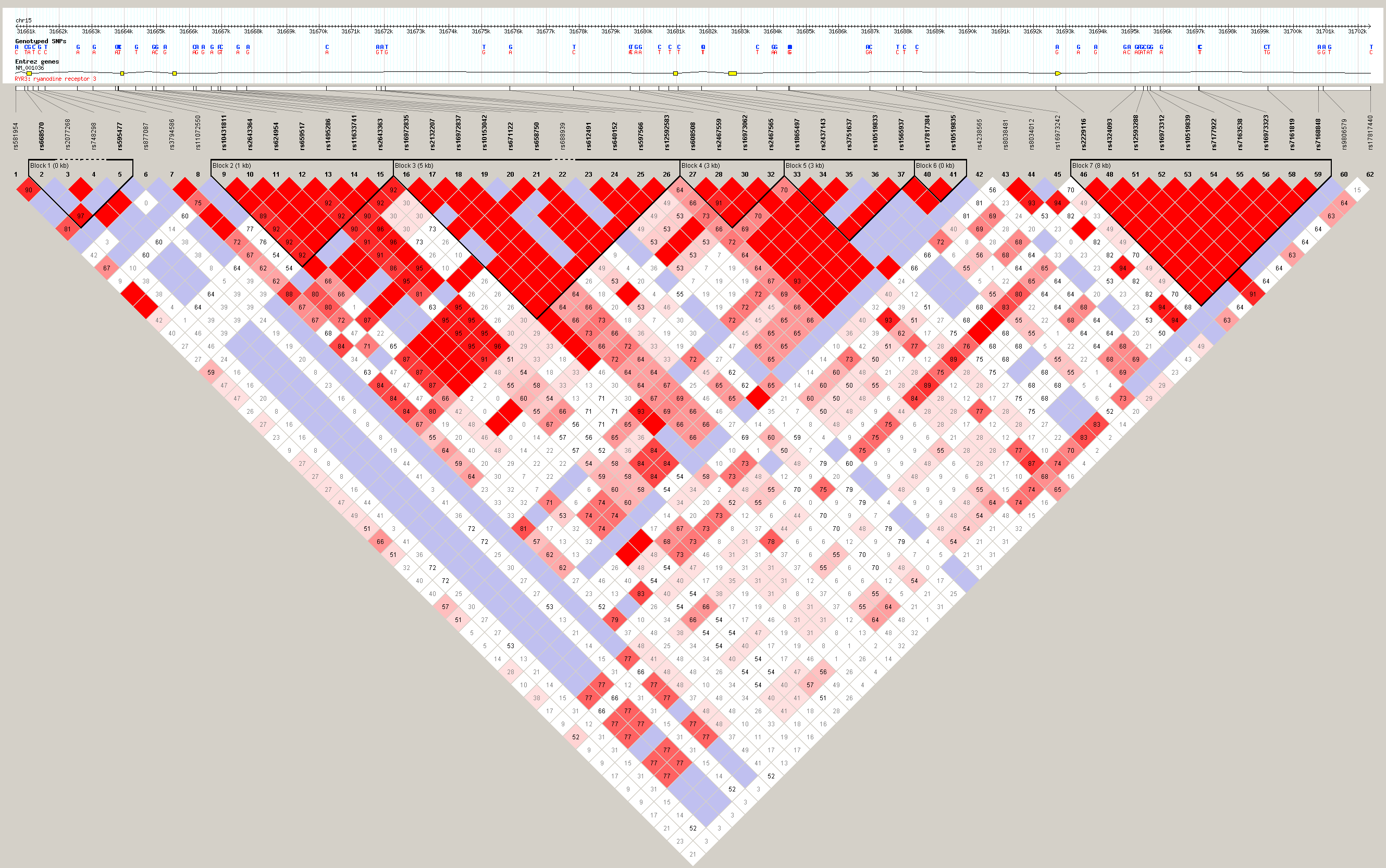

Supplement: Additional file 4: Figure S2 — The LD (D’) heatmap of the 31.1 Kb region in JPT. [file 1471-2261-14-6-S4.png]
